# Protective Effects of B Vitamins and Antioxidants on the Risk of Arsenic-Related Skin Lesions in Bangladesh

**DOI:** 10.1289/ehp.10707

**Published:** 2008-04-16

**Authors:** Lydia B. Zablotska, Yu Chen, Joseph H. Graziano, Faruque Parvez, Alexander van Geen, Geoffrey R. Howe, Habibul Ahsan

**Affiliations:** 1 Department of Epidemiology, Mailman School of Public Health, Columbia University, New York, New York, USA; 2 Department of Environmental Medicine, New York University School of Medicine, New York, New York, USA; 3 Department of Environmental Health Sciences, Mailman School of Public Health, Columbia University, New York, New York, USA; 4 Lamont-Doherty Earth Observatory of Columbia University, New York, New York, USA; 5 Department of Health Studies; 6 Department of Medicine; 7 Department of Human Genetics and; 8 Cancer Research Center, The University of Chicago, Chicago, Illinois, USA

**Keywords:** antioxidants, arsenic, Bangladesh, B vitamins, skin lesions

## Abstract

**Background:**

An estimated 25–40 million of the 127 million people of Bangladesh have been exposed to high levels of naturally occurring arsenic from drinking groundwater. The mitigating effects of diet on arsenic-related premalignant skin lesions are largely unknown.

**Objectives:**

The purpose of this study was to clarify the effects of the vitamin B group (thiamin, riboflavin, niacin, pyridoxine, and cobalamin) and antioxidants (vitamins A, C, and E) on arsenic-related skin lesions.

**Methods:**

We performed a cross-sectional study using baseline data from the Health Effects of Arsenic Longitudinal Study (HEALS), 2000–2002, with individual-level, time-weighted measures of arsenic exposure from drinking water. A total of 14,828 individuals meeting a set of eligibility criteria were identified among 65,876 users of all 5,996 tube wells in the 25-km^2^ area of Araihazar, Bangladesh; 11,746 were recruited into the study. This analysis is based on 10,628 subjects (90.5%) with nonmissing dietary data. Skin lesions were identified according to a structured clinical protocol during screening and confirmed with further clinical review.

**Results:**

Riboflavin, pyridoxine, folic acid, and vitamins A, C, and E significantly modified risk of arsenic-related skin lesions. The deleterious effect of ingested arsenic, at a given exposure level, was significantly reduced (ranging from 46% reduction for pyridoxine to 68% for vitamin C) for persons in the highest quintiles of vitamin intake.

**Conclusions:**

Intakes of B-vitamins and antioxidants, at doses greater than the current recommended daily amounts for the country, may reduce the risk of arsenic-related skin lesions in Bangladesh.

Inorganic arsenic occurring in various forms in the environment has been classified as a definite human carcinogen (group 1) since 1979 (International Agency for Research on Cancer 1980). Numerous studies conducted in Taiwan and South America found that exposure to inorganic arsenic from drinking water is associated with cancers of the bladder, kidneys, skin, and other organs and tissues (Cantor 1997; Ferreccio et al. 2000).

Similar to Taiwan, several countries in South Asia have high levels of naturally occurring arsenic in groundwater. In the early 1970s, the government of Bangladesh, with the support and financing of the United Nations Children’s Fund, promoted the digging of the tube wells to provide clean drinking water. In the late 1990s, evidence indicated that the groundwater, the main source of drinking water in Bangladesh, is contaminated by naturally occurring arsenic in 59 of the 64 districts of the country. An estimated 25–40 million of Bangladesh’s 127 million people have been exposed to levels frequently above the national limit of 50 ppb and often reaching levels as high as 800 ppb (British Geological Survey 2006).

Several studies have shown convincing evidence of the association between drinking arsenic-rich water and skin lesions, which are recognized as precursors of nonmelanoma skin cancer (Ahsan et al. 2006b; Guha Mazumder et al. 1998; Tondel et al. 1999). Recent studies conducted in South Asia have raised the possibility that antioxidants may modify the effects of water arsenic on the risk of skin lesions (Hsueh et al. 1995; Vahter 2000). Folate and cobalamin (vitamin B_12_) have been suggested to play an important role in the detoxification of ingested arsenic (Gamble et al. 2005b; Mitra et al. 2004; National Research Council 1999). Specifically, methylation of arsenic is a folic acid–dependent reaction catalyzed by a cobalamin-dependent enzyme (Gamble et al. 2005b; Zakharyan and Aposhian 1999). Steinmaus et al. (2005) showed that consumption of high levels of niacin (vitamin B_3_) was associated with arsenic methylation. Other studies showed some evidence that anti-oxidants such as vitamin A also play a role in diminishing arsenic toxicity (Chattopadhyay et al. 2002; Hsueh et al. 1995; Roychowdhury et al. 2003; Styblo and Thomas 2001). To date, few studies have evaluated the effects of diet specific to Bangladesh on the relationship between arsenic exposure from drinking water and skin lesions. McCarty et al. (2006) reported a significantly reduced risk associated with intake of fruits and canned goods, but were not able to assess the effects of individual food compounds.

The Health Effects of Arsenic Longitudinal Study (HEALS) was established to examine the health effects of arsenic exposure in Bangladesh. It is a population-based prospective cohort study in Araihazar, Bangladesh, with individual-level water arsenic measurements. We recently reported an increased dose-related risk of skin lesions in relation to arsenic exposure in this cohort (Ahsan et al. 2006b). As part of the investigation, we collected detailed information about the daily diet of all participants, using a food frequency questionnaire (FFQ) developed specifically for this population. The U.S. Department of Agriculture (USDA 2006) and Indian nutritional tables (Gopalan et al. 1996) were used to estimate the consumption of various vitamins and antioxidants. Here, using the baseline data of the HEALS cohort, we report the results of analyses aimed at clarifying the effects of the vitamin B group, including thiamin (vitamin B_1_), riboflavin (vitamin B_2_), niacin (vitamin B_3_), pyridoxine (vitamin B_6_), cobalamin (vitamin B_12_), and folic acid and antioxidants (vitamins A, C, and E) on the relationship between arsenic exposure and skin lesions.

## Materials and Methods

The detailed description of the study methods and participants of the HEALS cohort analyzed here has been published previously (Ahsan et al. 2006a, 2006b; Parvez et al. 2006). In brief, we identified and tested all 5,996 tube wells in the 25-km^2^ area of Araihazar, Bangladesh, and proceeded to recruit eligible cohort members from their 65,876 users. A total of 14,828 individuals met the following eligibility criteria: married and ≥ 18 years of age; resident in the study area for at least 5 years before recruitment; and user of one of the study wells for at least 3 years. Nineteen percent of the eligible individuals (*n* = 2,778) were not at home during study visits. Of the 12,050 individuals who were available and approached, 11,746 (97.5% response rate) were recruited to the cohort between October 2000 and May 2002. Informed consent was obtained from each eligible respondent who agreed to participate in the study. The study protocol and field procedures were approved by the Columbia University Institutional Review Board and by the Ethical Committee of the Bangladesh Medical Research Council.

### Measurement of arsenic exposure

Water samples from all tube wells were analyzed for arsenic concentrations by the graphite furnace arsenic absorption method, as described previously (van Geen et al. 2003). Detailed information about water consumption in the preceding years from questionnaires was used to construct the individual-level, time-weighted arsenic (TWA) exposure measure, taking into account both arsenic concentration and duration of water drinking from the index tube well (Ahsan et al. 2006a). Thus, TWA (in micrograms per liter) = ∑ *C_i_T_i_*/∑*T_i_*, where *C_i_* and *T_i_* denote the well arsenic concentration and drinking duration for the *i*th well, respectively.

All HEALS participants provided urine samples, which were used to estimate total urinary arsenic concentration by the graphite furnace arsenic absorption method, as described previously (Nixon et al. 1991). Urinary creatinine was analyzed using a method based on the Jaffe reaction (Slot 1965).

### Measurement of vitamins and antioxidants

The baseline interview collected information on diet using a semiquantitative 39-item FFQ designed to assess the long-term usual diet of cohort participants and described in detail elsewhere (Chen et al. 2004). Briefly, HEALS investigators, with help from local nutrition experts, first identified all the food items available at the village market in the study area. The FFQ was finalized after pilot testing to include common food items. Food items with intake frequencies less than once per month during the past year were deemed to be insignificant. We are confident that the food list covers > 90% of the typical daily diet in the study population (Chen et al. 2004), because the development of the food list was based on extensive pilot work and the diet in rural Bangladesh is relatively homogeneous and simple. Food diversity is limited by the low availability of tillable land. The average diet is almost entirely cereal-based, comprising very few highly processed food products. The use of dietary/nutritional supplements is rare in Bangladesh. The reasonable average daily intakes of energy computed from both the FFQ and a food diary (FD) gave reassurance on this aspect (Chen et al. 2004). Extensive work was done to assess the validity of the data collected from the FFQ, including two 7-day FDs for 189 randomly selected study subjects. The latter showed moderately good correlations for B vitamins with the FFQ; for example, energy-adjusted correlation coefficients with correction for within-individual variation were 0.46 for riboflavin, 0.39 for niacin, and 0.57 for cobalamin (Chen et al. 2004). Although large seasonal variations between the two FDs were observed for vitamins A and C, overall the FFQ was deemed an adequate tool to capture long-term intakes of common foods and nutrients in this population (Chen et al. 2004).

Nutrient values for foods in Bangladesh were not available. Therefore, we used the USDA National Nutrient Database for Standard Reference (USDA 2006) and an Indian food nutrient database (Gopalan et al. 1996) to estimate the intake of various nutrients from reported intake of foods. However, the Indian tables lack information on several important nutrients such as cobalamin and vitamin E. The correlations between all other nutrients discussed in the analysis estimated using both sets of tables were very good, in both males and females (Pearson’s correlation coefficients > 0.7 for all other nutrients; data not shown). Therefore, we present the results using the USDA tables. Individual B vitamins were highly correlated, with Pearson’s correlation coefficients ranging from 0.50 for niacin and folic acid to 0.98 for niacin and thiamin. All nutrient values were log-transformed to improve the normality of the distribution (Chen et al. 2004) and were adjusted for total energy using the residual method (Willett and Stampfer 1986). Average daily intakes of food groups and nutrients estimated by the FFQ in the study population are reported in the Appendix.

### Measurement of the outcome

A structured protocol was used to assess skin lesion presence during the clinical examination (Ahsan et al. 2006a). Briefly, to ensure uniformity in the clinical examination of skin lesions across the entire body, we instituted a structured protocol following the plan for quantitative assessment of the extent of body surface involvement in burn patients. The principle is based on dividing the entire body skin surface into 11 segments (e.g., front of arm, back of arm, face) and assigning percentages to each of them based on their size relative to the whole body surface. Each subject was examined by a physician of the same sex, who recorded presence or absence of the skin lesions and their size and shape. A total of 810 cases with skin lesions (hyperkeratosis and/or melanosis) were identified among 11,746 subjects; 714 (88.1%) of those with skin lesions were confirmed upon further clinical review [421 (337 males and 84 females) had melanosis only, and 293 (247 males and 46 females) had both hyperkeratosis and melanosis) (Ahsan et al. 2006b). When we evaluated the dose-dependent effect of arsenic separately for early-stage (melanosis) and late-stage (hyperkeratosis) skin lesions, the results were similar (Ahsan et al. 2006b). In the current analysis, the modifying effects of individual B vitamins and antioxidants did not differ by severity of skin lesions (data not shown), and therefore all subsequent analyses are presented for all skin lesions.

### Statistical analysis

Our first approach was to compare prevalence of skin lesions in the baseline cohort across the arsenic dose range. Others have suggested that the effects of many risk factors may vary among subgroups, depending on intake of dietary factors (Rothman and Greenland 1998; Willett 1998). Thus, our second statistical approach was to conduct categorical analysis of the data using unconditional logistic regression modeling for Bernoulli data (Breslow and Day 1987). In the model with water arsenic consumption, we estimated prevalence odds ratios (PORs) of skin lesions in quintiles of intake of various micronutrients. The cutpoints for these quintiles were chosen to evenly distribute all subjects. Given reasonable assumptions about the progression of such lesions to a clinically detectable state, the POR is a good approximation of the relative risk of skin lesions estimated from studies that do not involve screening (Ahsan et al. 2006b).

Most studies to date have concentrated on evaluating whether the relative risk associated with the main exposure of interest is constant across categories of the third variable, that is, under the assumption of multiplicative interaction (Rothman and Greenland 1998). We used an excess relative risk (ERR) model to estimate the ERR per unit of arsenic exposure. By adding 1.0 to the ERR, we obtained the relative risk per unit change in exposure. This risk model has the form


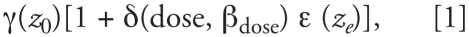


where γ , δ , and ɛ are functions of the background risks, the dose-related risks, and the risk-modifying factors, respectively; *z*_0_ is a possible independent factor; (*z_e_*) is a risk-modifying factor; and β dose is an arsenic dose parameter (ERR) to be estimated from the linear function of dose. Adjusted parameter estimates from this model can be directly (i.e., without exponentiation) interpreted as the increase in risk of skin lesions per unit dose of exposure in this population. Thus, any risk associated with arsenic exposure multiplies the background risk from independent risk factors other than arsenic, such as age and education, and the relationship between risk and arsenic dose is linear. Furthermore, the model gives effect estimates for the main effects of arsenic exposure, other independent risk factors, and potential modifying risk factors. The same model can be used to test effect modification on multiplicative scale efficiently (with fewer degrees of freedom), compared with methods based on conventional stratified analysis and the use of cross-product terms in unconditional logistic regression models.

Based on the literature review and previous analysis of the baseline data (Ahsan et al. 2006b; Argos et al. 2007), we specified a number of *a priori* confounders for the arsenic–skin lesion relationship such as sex, age (categorized into nine 5-year categories), body mass index (BMI; categorized into quintiles of an approximately equal number of subjects), education (< 1, 1–6, 6–11, and > 11 years), occupation, and television and land ownership (proxies of socioeconomic status). Several studies (e.g., Chen et al. 2006; McCarty et al. 2006), including our previous analysis of this cohort (Ahsan et al. 2006b), have shown that the use of tobacco products is associated with the risk of skin lesions independently of arsenic exposure.

We evaluated effects of consumption of various B vitamins (thiamin, riboflavin, niacin, pyridoxine, cobalamin, and folic acid) and antioxidants (vitamins A, C, and E) on arsenic-related skin lesions by two methods: we evaluated them for independent associations with skin lesions, and we used Equation 1 to evaluate their effect-modifying properties. When variables were evaluated as possible independent risk factors of skin lesions, an arsenic dose parameter was retained in the model to control for the main effects of dose and for possible confounding effects. Individual risk factors were retained in the model if they significantly improved the fit of the model, as evaluated by the likelihood ratio test comparing the deviances from the two nested models or if they changed the risk estimate by more than 10%. Urinary creatinine was included in all models analyzing the effects of urinary arsenic as a separate independent variable (Gamble and Liu 2005). To identify significant interaction, we used the trend test assessing relative risks across categories of micronutrient intake. In that case, the ordinal variable was used to represent increasing consumption categories.

Because the distributions of arsenic measurements were not normal, we constructed a standard unit of arsenic exposure to be used in the dose–response models whereby risk was estimated per unit of exposure defined as the interquartile range [difference between the 75th and 25th percentiles (Willett 1998)]. In our analysis, this unit was 131 μg/L for time-weighted water arsenic (range, 0.1–648.0 μg/L; mean, 98.2 μg/L) and 130 μg/L for urinary arsenic (range, 1–2273.0 μg/L; mean, 130.9 μg/L). Because the model is linear, the ERR per unit of dose for arsenic exposure could be converted to an ERR per 1-μg/L by dividing the ERR by 131.

All statistical tests were two-sided with a specified type I error of 0.05, yielding 95% confidence intervals (CIs) estimated by the maximum likelihood method. All analyses were performed using the GMBO module of the EPICURE statistical package (Preston et al. 1993).

### Study population

Of the total of 11,746 subjects recruited between October 2000 and May 2002, 522 (4.4%) did not provide urine samples and were excluded from this analysis. An additional 570 (4.8%) subjects were excluded either because there was not enough information in the questionnaire about their water-drinking habits to permit estimation of individual-level, time-weighted arsenic measures or because they did not undergo a physical examination for skin lesions status. Furthermore, 3 subjects had missing information on land ownership, an important *a priori–*defined confounder, and 23 subjects had very high levels of time-weighted arsenic measure, which were considered outliers. Thus, all analyses are based on the cohort of 10,628 subjects (90.5% of those recruited), 658 of whom had skin lesions and thus were considered eligible cases (92.2% of all cases).

## Results

In our study, approximately 45% of subjects consumed < 50 μg/L time-weighted water arsenic. Study subjects with skin lesions had significantly higher consumption compared with those who did not develop skin lesions (chi-square test, *p* < 0.001). Males were five times more likely to have skin lesions than females (POR = 5.4; 95% CI, 4.3–6.7) (Table 1). Cases tended to be older and, in general, risk of skin lesions increased monotonically with age (*p* < 0.001; data not shown). A number of risk factors have been examined as measures of socioeconomic status, but only education was a strong predictor of risk of skin lesions in this cohort. We observed a reduced POR for skin lesions (POR = 0.3; 95% CI, 0.2–0.6) for those with ≥ 11 years of education compared with those who had < 1 year of formal education. The association with BMI was not linear, but higher BMIs appeared to be inversely associated with skin lesions.

Table 2 presents the results of analyses investigating the independent effects of various B vitamins and other antioxidants on the risk of skin lesions after adjustment for arsenic exposure, total energy intake, and significant confounders identified in Table 1 (sex, age at risk, BMI, and education). For riboflavin, pyridoxine, vitamins A, C, and E, and folic acid, a reduced POR for skin lesions was observed for those in the highest quintiles of consumption (ranging from 46% for vitamin A to 60% for vitamin E) in risk of skin lesions.

**Figure 1:**
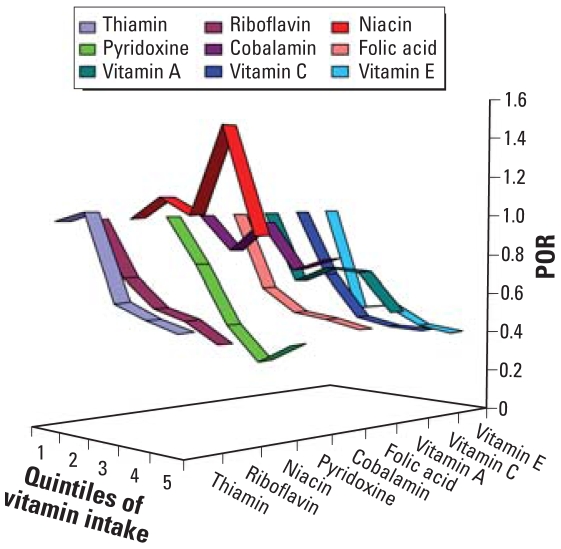
PORs for quintiles of vitamin intake from the categorical analysis.

Significant *p*-values of the linear trend test for riboflavin; pyridoxine; vitamins A, C, and E; and folic acid indicated a strong linear dose–response trend in the data. Figure 1 is a graphic representation of the adjusted PORs in Table 2. It is evident that consumption of most B vitamins, as well as antioxidants, was inversely associated with risks of skin lesions, as shown by downward sloping lines. The effects of thiamin, riboflavin, niacin, and cobalamin were less pronounced, and their estimates for various quintiles of consumption hovered around the null effect. Therefore, we did not evaluate the arsenic-modifying effects of these vitamins in the subsequent analyses.

In our cohort, intake of riboflavin strongly correlated with the intake of pyridoxine and folic acid (Pearson’s correlation coefficients 0.75 and 0.83, respectively; data not shown). When we investigated the combined independent effects of these three micro-nutrients by simultaneously entering them into the model, we observed slight attenuation of effect of arsenic-related skin lesions, but the reduction in risk in the upper quintiles of micronutrient consumption compared with the lowest quintile remained unchanged (data not shown).

Table 3 shows that after adjusting the background rates for sex, age at risk, education, BMI, and total energy consumption, those consuming water containing 131 μg/L time-weighted water arsenic had an ERR of skin lesions of 1.6 (95% CI, 1.0– 2.1); that is, for every 131-μg/L increase of time-weighted water arsenic, we observed a 2.6-fold higher risk of skin lesions. The effects of arsenic exposure were modified by consumption of vitamins. Table 3 shows that intake of riboflavin (model 2) was a strong modifier of the effects of arsenic. Those in the highest quintile of consumption had only 37% of the ERR (1.0 per 131 μg/L; 95% CI, 0.5–1.8) of those in the lowest quintile (ERR = 2.7 per 131 μg/L; 95% CI, 0.2–3.8). Similarly, intakes of folic acid (model 4) and vitamins A, C, and E (models 6–8) modified the arsenic-related skin lesion risks, with reductions of ERR estimates for skin lesions in the highest quintiles (compared with the lowest quintiles of micronutrient intake) ranging from 65 to 68%.

To estimate the combined effect of B vitamins on arsenic-related skin lesions, we repeated our analyses with simultaneous interaction terms for riboflavin, pyridoxine, and folic acid. We observed that those in the highest quintiles of consumption of all three micro-nutrients had only 37.8% of the ERR of those in the lowest quintiles of consumption for all three nutrients (ERR, 3.2 per 131 μg/L weighted water arsenic exposure) (model 5). Thus, on the basis of these results and those presented in Tables 2 and 3, it appears that the effects of the B vitamins are additive in nature.

We further investigated the effects of urinary arsenic, which was strongly correlated with the time-weighted arsenic exposure measure used in Table 3 (Pearson’s *r* = 0.47; data not shown). Similar to the analysis of the time-weighted arsenic exposure measure presented in Table 2, only riboflavin; pyridoxine; vitamins A, C, and E; and folic acid displayed an inverse effect on the risk of skin lesions (data not shown). Table 4 shows that, on average, ERRs associated with the urinary arsenic concentrations were > 3-fold higher than those associated with the water arsenic. Furthermore, riboflavin; pyridoxine; vitamins A, E, and C; and folic acid modify the effects of urinary arsenic. These effects are in the same direction (albeit stronger) as the effects observed for the time-weighted arsenic exposure measure in Table 3, with reductions in risk of skin lesions in the upper quintiles of vitamins intake ranging from 34 to 74%.

## Discussion

In this article we report the results of analysis of the baseline data from the prospective cohort study of the association between time-weighted water arsenic exposure and risk of skin lesions. Although skin lesions have been linked previously with exposure to arsenic-contaminated drinking water, there is a recognized lack of information on the modifying effects of local diet on this relationship (McCarty et al. 2006). To our knowledge, this is the first systematic analysis of the association between micronutrient intake and prevalence of arsenic-induced skin lesions.

We estimated an ERR of 1.6 (95% CI, 1.0–2.1) per 131-μg/L time-weighted water arsenic and an ERR of 4.91 (95% CI, 1.8–8.1) per 130-μg/L urinary arsenic concentration. Thus, those exposed to a dose equal to 50 μg/L water arsenic, the currently permissible arsenic limit in Bangladesh (and in the United States, until recently), had a 59% higher risk of skin lesions compared with those with dose zero. Even a small dose of water arsenic equal to the current water arsenic concentration limit in the United States of 10 μg/L carried a 12% increase in risk compared with those with zero doses.

We found that riboflavin; pyridoxine; vitamins A, C, and E; and folic acid were significant strong modifiers of the effects of ingested arsenic. Our results suggest that consumption of a diet rich in these vitamins and anti-oxidants can significantly reduce the harmful effects of water arsenic on the development of skin lesions. Those in the highest percentile of consumption had a significant reduction in risks, ranging from 46% (ERR decreased from 2.4 to 1.3) for pyridoxine to 68% (ERR decreased from 3.4 to 1.1) for vitamin C, at a given level of arsenic exposure (Table 3).

It is noteworthy that in our population, the consumption of riboflavin, pyridoxine, and vitamin A were significantly lower than the RDA values for India (Gopalan et al. 1996) (data not shown). For example, the recommended daily amount for both males and females for pyridoxine is 2 mg, but our cohort members consumed, on average, 0.66 and 0.61 mg/day (males and females, respectively). Vitamin consumption was better for folic acid and vitamin C. In our study, the potential protective modifying effects of these vitamins were restricted within the medium and upper quartiles of consumption. Thus, it appears that beneficial effects of riboflavin; pyridoxine; vitamins A, C, and E; and folic acid are associated with consumption of amounts greater than the current recommended daily amounts for the population.

Among the major strengths of the present study are use of individual-level, time-weighted water arsenic measures, micronutrient measure from a validated instrument, and the large size of the study population. Caution should be exercised while interpreting these findings, as they are based on numerous comparisons and could be due to chance. In addition, our analyses are based on the prevalent skin lesions. We are continuing follow-up of the cohort and will analyze the data on the incident skin lesion identified during the first and second two-yearly follow-up visits. The use of FFQ introduced inevitable measurement errors. If present, this measurement error would attenuate the association (bias it toward the null effect). Future studies with biochemical measures of nutrient intakes, especially for vitamins A, C, and E, are needed to further evaluate the effects of these nutrients.

The evidence on the health effects of arsenic from food is limited. Although several studies have documented arsenic contents in rice collected in arsenic-affected areas (Misbahuddin 2003; Roychowdhury et al. 2003), the bioavailability of arsenic from rice and other food items is not known. The findings of the present study would not be affected by the potential role of arsenic in rice, as rice is not the main source of the nutrients found to have a modifying effect. If arsenic in rice indeed has an effect on skin lesions, the measurement error in total arsenic exposure may lead to a bias toward the null for the observed dose–response relationship. Furthermore, arsenic in food is considered to be present largely in organic forms, and the content depends on the arsenic concentration in the soil as well as in the water used for washing, cooking, and irrigation (Roychowdhury et al. 2003). Future studies are needed to consider the potential role of arsenic in food items. Finally, many participants drank water from the same well (59.2% of study participants drank from a well with ≥ 3 subjects/well, 31.9% from a well with two study subjects/well, and the remaining 8.9% of subjects from individual wells), making well water arsenic concentration a shared characteristic. These correlated errors arising from shared wells would affect the width of the CIs, but should not affect the magnitudes or directions of the point estimates.

Methylation of arsenic, a hypothesized detoxification pathway, requires the conversion of *S*-adenosylmethionine to *S*-adenosylhomocysteine and depends partly on the one-carbon metabolism in which riboflavin, pyridoxine, cobalamin, and folic acid all play a role (Selhub 2002). Previous studies have shown that intake of vitamins influences the efficiency of arsenic methylation (McCarty et al. 2006; Steinmaus et al. 2005). Our findings that riboflavin, pyridoxine, and folic acid modified the risk of skin lesions are consistent with the hypothesis that individuals with insufficient intakes of nutrients related to arsenic metabolism are more susceptible to the health effect of arsenic exposure. These findings further suggest that riboflavin, pyridoxine, and folic acid may play a more important role in modifying arsenic toxicity.

Although folic acid is readily available in many food items, its deficiency is not uncommon, primarily because naturally occurring folates are highly susceptible to oxidative degradation, for example, during cooking (Gamble et al. 2005b). We reported previously that there is a high prevalence of folate deficiency and hyperhomocysteinemia in Araihazar, Bangladesh (Gamble et al. 2005a) and that these conditions are associated with reduced arsenic methylation (Gamble et al. 2005b).

Intracellular antioxidants such as vitamins A, C, and E decrease arsenic toxicity by reversing disturbances in lipid peroxidation, generation of nitric oxide, reactive oxygen species, and apoptosis initiated by arsenic metabolites (Chattopadhyay et al. 2002; National Research Council 1999). β-Carotene may also scavenge free-radical species (Krinsky 1989). Previous studies have shown that low serum levels of carotene modified the effect of arsenic exposure on the risk of ischemic heart disease (Hsueh et al. 1998). Several studies have found that prevalence of vitamin A deficiency is high in Bangladesh (Ahmed 1999), possibly explaining the important modifying effect of vitamin A observed in this study. Given that vitamin A consumption in the Bangladeshi diet is mostly from plant sources, the correlation between beta carotene intake and vitamin A in our study was nearly perfect (*r* = 0.99), explaining the similarity of modifying effects of vitamin A and beta carotene on arsenic-related skin lesions.

Overall malnutrition, defined as either a general calorie deficit or a diet adequate in calories but nutritionally poor, is an important cofactor in arsenic poisoning affecting the timing and the intensity of arsenic-related problems. Guha Mazumder et al. (1998) showed that subjects with lower BMIs had higher prevalence of arsenic skin lesions compared with subjects with similar arsenic exposures but higher BMIs. Similarly, in our previous analysis of this cohort, we showed that study subjects with lower BMIs were at increased risk of skin lesions (Ahsan et al. 2006b). Current analysis indicates that BMI has an independent effect on the development of skin lesions, and thus, subjects with lower consumption of vitamins and antioxidants had higher risk of skin lesions, indicating significant effects of individual nutrients beyond what can be explained by general calorie intake.

Findings on the modifying effect of sunlight exposure on arsenic-related skin lesions have been described elsewhere (Chen et al. 2006). Because women in Bangladesh universally wear traditional dresses that almost completely cover the skin of their trunk, sunlight exposure of female respondents was considered minimal and therefore was not assessed in the study. In men, we observed an additive effect of higher arsenic exposure and excessive sunlight exposure, such that the risk of skin lesions associated with any given level of arsenic exposure was greater in males with excessive sun exposure. In the present analysis, we empirically assessed whether sunlight exposure is a potential confounder. Adjustment for sunlight exposure did not change the effect estimate and therefore we did not include it in the model.

## Conclusion

The results of this study strongly suggest that consumption of foods rich in vitamins such as riboflavin; pyridoxine; vitamins A, C, and E; and folic acid may influence the relationship between exposure to water arsenic and subsequent risk of skin lesions. Those in the highest quintiles of consumption had significant reductions in risks for skin lesions associated with arsenic exposure. The observed modifying effects were associated with consumption of nutrients at doses that are higher than the current recommended daily amounts for the country. These findings support the hypothesis that nutrients relevant to arsenic metabolism and antioxidant nutrients may modify the risk of arsenic-related skin lesions in Bangladesh. Future studies looking at that influence of micronutrients on risk of incident arsenic-related skin lesions are necessary to clarify the nature of the association. Intervention studies are also needed to determine whether dietary supplementation may mediate health effects of arsenic exposure.

## Figures and Tables

**Table 1:** Sociodemographic characteristics of the study participants.

Variable	Cases [no. (%)]	Noncases [no. (%)]	Adjusted POR^a^ (95% CI)	*p*-Value^b^
Water arsenic (μg/L)				
0.1–49.9	169 (25.7)	4,539 (45.5)		
50.0–99.9	133 (20.2)	1,833 (18.4)		
100.0–199.9	166 (25.2)	2,077 (20.8)		
200.0–648.0	190 (28.9)	1,521 (15.3)		
Sex				
Female	122 (18.5)	5,978 (60.0)	1	< 0.001
Male	536 (81.5)	3,992 (40.0)	5.4 (4.3–6.7)	
Age (years)				
< 25	13 (2.0)	1,020 (10.2)	1	< 0.001
25–29	24 (3.6)	1,630 (16.3)	0.8 (0.4–1.5)	
30–34	62 (9.4)	1,803 (18.1)	1.5 (0.8–2.7)	
35–39	101 (15.3)	1,782 (17.9)	2.0 (1.1–3.7)	
40–44	114 (17.3)	1,296 (13.0)	3.0 (1.7–5.5)	
45–49	124 (18.8)	1,187 (11.9)	3.6 (2–6.5)	
50–54	96 (14.6)	675 (6.8)	4.0 (2.2–7.4)	
55–59	77 (11.7)	393 (3.9)	4.3 (2.3–8)	
≥ 60	47 (7.1)	184 (1.8)	4.6 (2.4–9)	
BMI				
< 17.2	177 (26.9)	1,929 (19.3)	1	0.065
17.2–18.6	165 (25.1)	2,092 (21.0)	1.0 (0.8–1.2)	
18.6–20.0	133 (20.2)	1,939 (19.4)	1.1 (0.8–1.3)	
20.0–22.3	102 (15.5)	2,074 (20.8)	0.8 (0.6–1.1)	
≥ 22.3	81 (12.3)	1,936 (19.4)	0.8 (0.6–1.1)	
Education (years)				
< 1	345 (52.4)	4,429 (44.4)		< 0.001
1–6	192 (29.2)	2,904 (29.1)	0.9 (0.7–1.0)	
6–11	106 (16.1)	2,228 (22.3)	0.6 (0.5–0.8)	
≥ 11	15 (2.3)	409 (4.1)	0.3 (0.2–0.6)	

a Adjusted for time-weighted arsenic, sex, age at risk, BMI, education, and total energy intake.

b Likelihood ratio test comparing the nested model with a model with additional variable.

**Table 2:** PORs and 95% CIs for the effects of B vitamins and antioxidants on risk of skin lesions.

Vitamin, energy-adjusted values (quintiles)	Adjusted POR^a^	95% CI	*p*-Value^b^
Thiamin (mg/day)			
< 1.62	1		0.162
1.62–1.91	1	0.7–1.7	
1.92–2.19	0.6	0.4–1.2	
2.20–2.50	0.6	0.3–1.2	
≥ 2.51	0.5	0.2–1.2	
Riboflavin (mg/day)			
< 0.69	1		< 0.001
0.69–0.82	0.7	0.6–1.0	
0.83–0.96	0.6	0.4–0.8	
0.97–1.16	0.6	0.4–0. 8	
≥ 1.17	0.5	0.3–0.7	
Niacin (mg/day)			
< 23.48	1		0.365
23.48–28.67	1.1	0.6–2.0	
28.68–32.44	1.0	0.5–2.1	
32.45–37.97	1.5	0.6–3. 6	
≥ 37.98	1.0	0.3–2.6	
Pyridoxine (mg/day)			
< 2.72	1		0.001
2.72–3.17	0.8	0.5–1.1	
3.18–3.65	0.5	0.3–0. 8	
3.66–4.18	0.3	0.2–0.6	
≥ 4.19	0.4	0.2–0.8	
Cobalamin (mg/day)			
< 0.84	1		0.200
0.84–1.30	0.8	0.6–1.1	
1.31–1.80	1.0	0.8–1.3	
1.81–2.53	0.8	0.6–1.0	
≥ 2.54	0.8	0.6–1.1	
Folic acid (μg/day)			
< 196.64	1		< 0.001
196.64–240.63	0.6	0.4–0.8	
240.64–283.99	0.5	0.4–0.7	
284.00–351.60	0.5	0.3–0.7	
≥ 351.61	0.5	0.3–0.6	
Vitamin A (mg/day)			
< 2298.48	1		< 0.001
2298.48–3503.45	0.7	0.5–0.8	
3503.46–4886.75	0.7	0.5–1.0	
4886.76–7113.05	0.7	0.5–0.9	
≥ 7113.06	0.5	0.4–0.7	
Vitamin C (mg/day)			
< 52.57	1		< 0.001
52.57–71.62	0.7	0.5–0.9	
71.63–92.67	0.5	0.3–0.6	
92.68–126.16	0.4	0.3–0.6	
≥ 126.17	0.4	0.3–0.6	
Vitamin E (mg/day)			
< 4.06	1		< 0.001
4.06–4.79	0.5	0.3–0.7	
4.80–5.52	0.5	0.4–0.7	
5.53–6.40	0.4	0.3–0.6	
≥ 6.41	0.4	0.3–0.6	

a Adjusted for time-weighted water arsenic, sex, age at risk, BMI, education, and total energy intake.

b Test of homogeneity of PORs across quintiles of micronutrient intake.

**Table 3:** Modification of effect of water arsenic on skin lesions by B vitamins and antioxidants.

Model^a^	Variable, energy-adjusted values (quintiles)	ERR^b^	95% CI	*p*-Value^c^
1	Water arsenic (μg/L)	1.6	1.0–2.1	
2	Riboflavin (mg/day)			
	< 0.69	2.7	0.2–3.8	0.010
	0.69–0.82	2.0	1.3–3.1	
	0.83–0.96	1.3	0.8–2.2	
	0.97–1.16	1.4	0.8–2.3	
	≥ 1.17	1.0	0.5–1.8	
3	Pyridoxine (mg/day)			
	< 2.72	2.4	1.2–3.6	0.054
	2.72–3.17	2.2	1.3–3.7	
	3.18–3.65	1.3	0.7–2.5	
	3.66–4.18	0.8	0.4–1.8	
	≥ 4.19	1.3	0.7–2.7	
4	Folic acid (μg/day)			
	< 196.64	3.2	1–4.4	< 0.001
	196.64–240.63	1.8	1.2–2.8	
	240.64–283.99	1.3	0.8–2.1	
	284.00–351.60	1.2	0.7–1.9	
	≥ 351.61	1.1	0.7–1.8	
5	Combined model: riboflavin (mg)/pyridoxine (mg)/folic acid (μg)			
	< 0.69/< 2.72/< 196.64	3.2	1.6–4.9	0.005
	0.69–0.82/2.72–3.17/196.64–240.63	2.2	0.9–3.5	
	0.83–0.96/3.18–3.65/240.64–283.99	1.1	0.3–1.9	
	0.97–1.16/3.66–4.18/284.00–351.60	0.9	0.3–1.6	
	≥ 1.17/≥ 4.19/≥ 351.61	1.2	0.5–2.0	
6	Vitamin A (mg/day)			
	< 2298.48	2.6	1.7–3.6	< 0.001
	2298.48–3503.45	1.2	0.8–2.0	
	3503.46–4886.75	1.7	1.1–2.6	
	4886.76–7113.05	1.7	1.1–2.6	
	≥ 7113.06	0.9	0.5–1.5	
7	Vitamin C (mg/day)			
	< 52.57	3.4	2.3–4.6	< 0.001
	52.57–71.62	2.0	1.4–2.9	
	71.63–92.67	1.2	0.7–1.9	
	92.68–126.16	1.1	0.7–1.7	
	≥ 126.17	1.1	0.7–1.8	
8	Vitamin E (mg/day)			
	< 4.06	3.4	1.9–4.9	0.002
	4.06–4.79	1.3	0.8–2.2	
	4.80–5.52	1.5	0.9–2.5	
	5.53–6.40	1.2	0.6–2.1	
	≥ 6.41	1.2	0.6–2.1	

a Models adjusted for sex, age at risk, BMI, education, and total energy intake.

b ERR of arsenic-related skin lesions per 131-μg/L time-weighted water arsenic for quintiles of micronutrient intake.

c Trend test using ordinal variable to represent increasing quintiles of micronutrient intake.

**Table 4:** Modification of effect of urinary arsenic on skin lesions by B vitamins and antioxidants.

Model^a^	Variable, energy-adjusted values (quintiles)	ERR^b^	95% CI	*p*-Value^c^
1	Urinary arsenic (μg/L)	4.9	1.8–8.1	
2	Riboflavin (mg/day)			
	< 0.69	9.0	3.4–14.5	< 0.001
	0.69–0.82	5.6	3.9–7.8	
	0.83–0.96	4.3	3.0–6.3	
	0.97–1.16	3.6	2.4–5.5	
	≥ 1.17	2.8	1.7–4.4	
3	Pyridoxine (mg/day)			
	< 2.72	10.7	2.7–18.7	< 0.001
	2.72–3.17	7.6	5.0–11.7	
	3.18–3.65	4.2	2.5–7.2	
	3.66–4.18	2.8	1.5–5.5	
	≥ 4.19	4.0	2.0–7.9	
4	Folic acid (μg/day)			
	< 196.64	10.6	3.8–17.5	< 0.001
	196.64–240.63	6.0	4.3–8.2	
	240.64–283.99	4.9	3.4–6.9	
	284.00–351.60	4.2	2.9–6.0	
	≥ 351.61	3.9	2.7–5.7	
5	Vitamin A (mg/day)			
	< 2298.48	8.1	2.8–13.5	< 0.001
	2298.48–3503.45	4.9	3.5–6.7	
	3503.46–4886.75	5.8	4.2–7.8	
	4886.76–7113.05	5.4	3.9–7.3	
	≥ 7113.06	3.4	2.4–4.9	
6	Vitamin C (mg/day)			
	< 52.57	12.2	4.2–20.1	< 0.001
	52.57–71.62	7.6	5.6–10.0	
	71.63–92.67	4.1	2.9–5.8	
	92.68–126.16	4.4	3.2–6.2	
	≥ 126.17	4.1	2.9–6.0	
7	Vitamin E (mg/day)			
	< 4.06	11.6	3.9–19.4	< 0.001
	4.06–4.79	4.6	3.1–6.9	
	4.80–5.52	4.8	3.0–7.3	
	5.53–6.40	3.5	2.1–5.7	
	≥ 6.41	3.3	1.9–5.6	

a Estimates of effect adjusted for sex, age at risk, BMI, education, urinary creatinine, and total energy intake.

b ERR of arsenic-related skin lesions per 130-μg/L urinary arsenic for quintiles of micronutrient intake.

c Trend test using ordinal variable to represent increasing quintiles of micronutrient intake.

## Appendix

**Appendix:** Average daily intakes of food groups and nutrients estimated by the FFQ.^a^

Food group	Food item	Mean ± SD (g/day)	Nutrients^b^	Mean ± SD
Milk	Milk	25.44 ± 45.51	Energy (kJ)	2285.94 ± 621.23
Rice	Puffed rice	19.56 ± 28.31	Total fat (g)	24.22 ± 9.35
	Steamed rice	1568.27 ± 524.14	Monounsaturated fat (g)	8.11 ± 3.52
	Water rice	26.09 ± 78.85	Polyunsaturated fat (g)	6.75 ± 1.9
Bread	Wheat bread (brown)	17.41 ± 46.65	Saturated fat (g)	5.81 ± 3.17
Tea	Tea	63.68 ± 173.67	Cholesterol (mg)	101.92 ± 89.76
Meat	Beef or mutton	14.89 ± 25.69	Protein (g)	72.84 ± 21.3
Beans	Lentil	78.13 ± 84.81	Carbohydrate (g)	447.17 ± 124.86
	Beans (scarlet runner)	36.42 ± 24.99	Fiber (g)	43.49 ± 11.79
Potato	Potato	110.06 ± 91.46	Thiamin (mg)	2.09 ± 0.55
	Sweet potato	4.81 ± 8.12	Riboflavin (mg)	0.95 ± 0.33
Poultry	Poultry (duck or fowl)	3.54 ± 11.48	Niacin (mg)	31.14 ± 8.55
Vegetables	Cabbage	9.24 ± 14.62	Pyridoxine (mg)	3.49 ± 0.97
	Cauliflower	21.89 ± 17.44	Folate (μg)	281.08 ± 111.23
	Eggplant	13.45 ± 25.39	Cobalamin (mg)	1.81 ± 1.26
Squash	Ghosala	4.61 ± 7.89	Retinol equivalents (mg)	294.36 ± 221.58
	Bitter gourd	8.35 ± 11.64	Vitamin C (mg)	96.05 ± 64.68
	Bottle gourd	19.06 ± 21.77	Vitamin E (mg)	5.35 ± 1.66
	Ridge gourd	4.09 ± 7.3	Calcium (mg)	367.6 ± 126.52
	Snake gourd	6.74 ± 8.24	Copper (mg)	2.4 ± 0.64
	Parwar	6.72 ± 10.89	Iron (mg)	12.78 ± 3.81
	Okra	8.00 ± 10.66	Magnesium (mg)	854.44 ± 235.16
	Green papaya	6.16 ± 16.08	Manganese (mg)	16.51 ± 4.73
	Pumpkin	3.03 ± 7.49	Potassium (mg)	2701.95 ± 950.83
	Radish	14.12 ± 17.2	Selenium (mg)	189.93 ± 53.38
	Spinach	17.08 ± 28.38	Sodium (mg)	434.12 ± 285.15
	Spinach stalks	12.76 ± 17.55	Zinc (mg)	13.7 ± 3.88
	Tomato	52.82 ± 47.48		
	Yam	9.44 ± 15.94		
	Jack fruit	17.14 ± 25.37		
	Mango	30.76 ± 52.2		
	Watermelon	4.38 ± 13.43		
Eggs	Eggs (hen eggs)	7.85 ± 15.47		
Fish	Big fish (fresh water)	22.68 ± 23.5		
	Dry fish	1.96 ± 5.22		
	Salted fish	0.37 ± 2.24		
	Small fish (fresh water)	29.06 ± 28.12		

a For 10,628 study participants included in the analysis.

b USDA nutrient database (USDA 2006).
